# The Clinical Relevance of FTO as a Demethylase Beyond Cancer: Molecular Mechanisms and Therapeutic Opportunities

**DOI:** 10.14336/AD.2025.0916

**Published:** 2026-09-10

**Authors:** Cun-yang Guo, Yi-fei Du, Hui-cong Yan, Xin-ming Fan, Fang-qi Tian, Ping Xu

**Affiliations:** ^1^School of Public Health, Binzhou Medical University, Yantai, Shandong, 264003, China.; ^2^Laboratory of Radiation-induced Diseases and Molecule-targeted Drugs, School of Food and Biomedicine, Zaozhuang University, Zaozhuang, Shandong, 277160, China.; ^3^School of Public Health, Shandong Second Medical University, Weifang, Shandong, 261000, China.; ^4^Department of Radiotherapy, Zaozhuang Municipal Hospital, Zaozhuang, Shandong, 277100, China

**Keywords:** FTO, RNA demethylation, epigenetics, molecular docking, bibliometric analysis

## Abstract

The fat mass and obesity-associated (FTO) gene is a key gene that has been linked to obesity and metabolic regulation. FTO single nucleotide polymorphisms (SNPs) significantly contribute to the pathophysiology of various multisystem diseases via epigenetic mechanisms. Although FTO has been extensively reviewed in the context of cancer, a comprehensive evaluation of its role in non-malignant diseases is currently lacking. This review aimed to systematically assess the molecular functions of FTO in the context of diseases other than cancer based on recent insights from the literature. Relevant studies were retrieved by systematically searching the PubMed database to explore the mechanisms through which FTO acts as a mediator of demethylation, its biological activities, and its functional roles in a spectrum of non-oncologic diseases. To explore pharmacological interactions, AutoDockTools 1.5.7 was used to simulate binding interactions between FTO and conventional therapeutic agents. Additionally, we conducted a bibliometric analysis using VOSviewer to visualize the frequency and co-occurrence of FTO-related terms, thereby helping to map research trends and knowledge gaps in the field. FTO regulates gene expression by modulating RNA methylation, particularly through the demethylation of N6-methyladenosine (m^6^A), thereby influencing RNA splicing, stability, and translation. This regulatory activity plays a major role in processes such as inflammation and fibrosis. Dysregulation of FTO has been implicated in several non-malignant diseases, including metabolic disorders, neurological diseases, and cardiovascular conditions. Computational docking studies showed that FTO exhibited strong binding affinity with two drugs and moderate affinity with eight others. Bibliometric analysis revealed high-frequency keywords and visualized research hotspots pertaining to FTO, providing valuable insight into current areas of scientific interest and potential future directions of study. FTO functions as a key epigenetic regulator in non-cancerous diseases and represents a promising biomarker and therapeutic target. Our findings underscore the importance of FTO-drug interactions and suggest that small-molecule FTO modulators may hold therapeutic value for managing a variety of non-oncologic conditions.

## 1. Introduction

Since its identification as a body mass index (BMI)-associated gene in 2007 [1], the fat mass and obesity-associated protein (FTO) have been increasingly established as a key regulator of diverse physiological and pathological processes [2-4]. While many studies have probed the molecular roles of FTO in oncogenesis, its functions beyond cancer remain incompletely understood, particularly in the context of metabolic dysregulation, neurological degeneration, apoptosis, and cardiovascular repair. This review provides an overview of recent findings detailing the ability of FTO to orchestrate pathological networks in non-neoplastic disorders, with a particular focus on how its RNA N6-methyladenosine (m6A) demethylase activity shapes the epigenetic regulatory networks that interconnect metabolic, neurological, and cardiovascular disease pathways.

In the context of metabolic disorders, FTO influences the expression of key genes including peroxisome proliferator-activated receptor gamma (PPAR-γ) and adrenoceptor β2 (ADRB2) through m6A-dependent mechanisms. This regulation disrupts adipogenesis, impairs insulin signaling, and contributes to enhanced susceptibility to obesity and type 2 diabetes mellitus [1]. Evidence suggests that the demethylase activity of FTO can govern mRNA stability, thereby shaping lipogenic regulatory gene expression to modulate lipid biosynthesis and adipocyte function. Similarly, in hepatic metabolism, FTO governs the expression of transcriptional regulators central to lipid homeostasis, ultimately affecting clinical markers such as serum lipid profiles [2,5].

In the central nervous system, FTO polymorphisms associated with an increased disease risk have been implicated in the progression of Alzheimer’s disease (AD), notably by enhancing β-amyloid deposition and Tau hyperphosphorylation [3]. In models of Parkinson’s disease (PD), loss of FTO activity worsens mitochondrial dysfunction and facilitates α-synuclein (α-syn) aggregation in dopaminergic neurons, accelerating neurodegeneration [6]. Additionally, FTO plays a role in cerebrovascular integrity, where its regulation of m6A dynamics contributes to vascular repair post-stroke. Conversely, metabolic comorbidities that elevate FTO expression may exacerbate ischemic vulnerability through FTO-mediated pathways [7].

In cardiovascular biology, FTO acts both indirectly via its effects on obesity-associated systemic metabolic inflammation and directly through its effects on vascular and myocardial cells. Of particular interest, FTO has been shown to exert cardioprotective effects by attenuating post-infarction cardiac fibrosis through the m6A-dependent suppression of pro-fibrotic gene expression, thereby limiting the deposition of collagen [4].

This review provides a systematic overview of the roles that FTO plays in non-neoplastic diseases, highlighting its molecular properties, enzymatic mechanisms of action, regulatory functions, and disease-specific implications. In addition to summarizing key experimental and clinical findings, we discuss recent advances in drug-target docking studies targeting FTO and apply bibliometric techniques to visualize research trends and thematic concentrations across FTO-related research.

## 2. FTO Structure and Function

**Figure 1.**
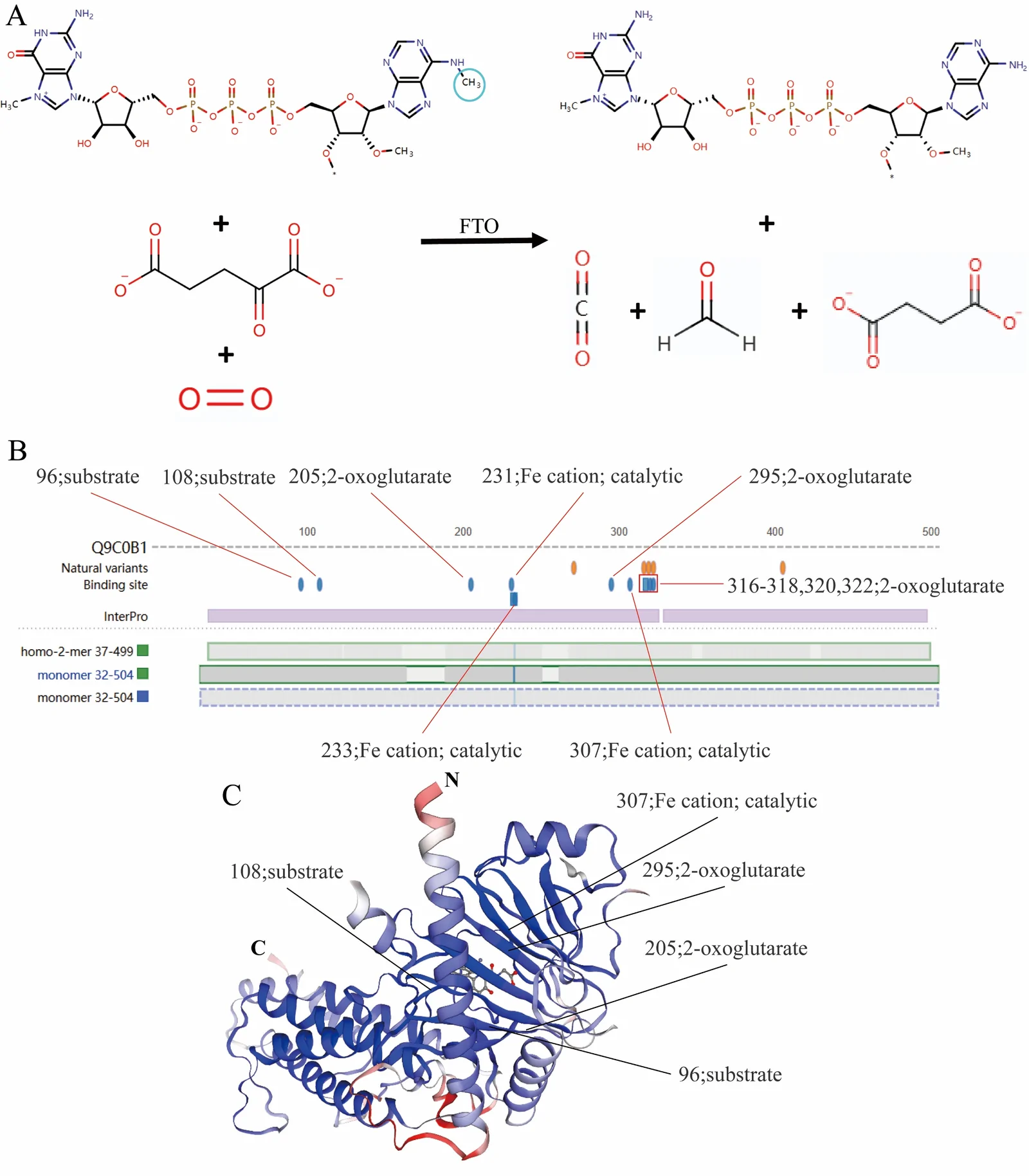
**Structure and Function of FTO**. (**A**) The demethylation process of removing m6A modification from RNA; (B) The full length and structural domains of FTO; (C) Three-dimensional structure of FTO (PDB ID: 4QHO).

FTO functions predominantly as an m6A RNA demethylase, targeting N6-methyladenosine, which is the most common internal modification found in eukaryotic messenger RNA. Through the selective removal of m6A marks, FTO alters the structural conformation, stability, and translational efficiency of RNA transcripts, thereby playing a critical role in post-transcriptional gene regulation and protein synthesis [8]. The demethylation reaction catalyzed by FTO involves the oxidative removal of the methyl group from a modified mRNA substrate. Specifically, FTO acts on 5′-end (N7-methyl-5′-triphosphoguanosine)-(N6,2′-O-dimethyladenosine) mRNA in the presence of molecular oxygen and 2-oxoglutarate. This enzymatic process yields 5′-end (N7-methyl-5′-triphosphoguanosine)-(2′-O-methyladenosine) mRNA, accompanied by the production of succinate, carbon dioxide, and formaldehyde as byproducts (Fig. 1A). The human FTO protein consists of 505 amino acids and is organized into three distinct domains: an N-ketoglutarate (NOG) domain (residues 1-31), a larger N-terminal domain (NTD, residues 32-326), and a C-terminal domain (CTD, residues 327-505) [9]. The FTO protein consists of two primary structural domains that govern its function and disease associations. These include regions harboring natural variants linked to clinical phenotypes, and active sites essential for enzymatic catalysis. Genetic variants in FTO have been associated with conditions such as microcephaly, developmental delay, facial dysmorphism, hypotonia, behavioral abnormalities, and reduced demethylase activity. The enzyme’s catalytic core includes substrate-binding residues (positions 96 and 108), cofactor-binding sites essential for enzymatic activity (positions 205, 295, 316-318, 320, and 322), and iron-binding sites required for oxidative demethylation (His231, Asp233, and His307) (Fig. 1B). Structurally, the N-terminal domain (NTD) of FTO features a conserved double-stranded β-helix (DSBH) fold, which forms a twisted β-sheet structure known as a jelly roll motif, stabilized by additional β-strands (β3 and β4) [10]. Within the NTD, Fe²^+^ ions are coordinated by conserved residues (His231, Asp233, His307), while α-ketoglutarate (NOG) forms salt bridges with Arg316 and Arg322. In contrast, the C-terminal domain (CTD) adopts a three-helix bundle that interacts closely with the NTD to maintain conformational stability and regulate enzymatic function [9] (Fig. 1C).

## 3. Molecular Mechanisms and Recent Research Trends

### 3.1 Molecular Mechanisms of Action for FTO

FTO was the first identified enzyme capable of demethylating RNA m6A modifications. It plays a central role in post-transcriptional gene regulation via Fe(II)/α-ketoglutarate (α-KG)-dependent oxidative demethylation. FTO preferentially recognizes the RRACH sequence motif in RNA, mediating m6A removal and thereby affecting mRNA splicing, translation, and stability [8]. Its activity extends beyond mRNA to include long non-coding RNAs (lncRNAs) and transfer RNAs (tRNAs). The localization of FTO within the cell is dynamic: nuclear FTO is primarily involved in snRNA demethylation, while cytoplasmic FTO regulates the expression of metabolic genes such as SIRT1 and PPAR-γ by controlling mRNA stability [11].

The involvement of FTO in non-cancerous diseases is multifaceted (see Table 1). In metabolic disorders, FTO modulates energy balance and adipogenesis. For example, the rs9939609 SNP promotes adipocyte differentiation and suppresses brown fat formation by demethylating the mRNAs encoding IRX3 and IRX5, contributing to obesity and insulin resistance [12]. In type 2 diabetes, FTO influences glucose metabolism by modulating the expression of GLUT2 and insulin signaling genes through m^6^A demethylation [13]. Neurologically, FTO affects brain development and synaptic plasticity. In stroke models, FTO supports neuronal survival and regeneration by demethylating Bcl2 mRNA [14]. In cardiovascular disease, FTO contributes to pathogenesis by regulating cholesterol metabolism and vascular inflammation. Its overexpression enhances foam cell formation and promotes atherogenesis by demethylating PPAR-γ and ABCA1 transcripts [15].

**Table 1:** Biological Functions and Targets of FTO in Specific Diseases.

Disease Category	Disease	Function	Targets	Mechanism	FTO level	Refs
**Metabolic Disease**	Obesity	Obesity phenotype	rs9939609, rs1421085	mRNA augmentation	Up	[16]
	Lipid Metabolism	Lipogenesis	FAS, SCD, ACC1	mRNA augmentation	Up	[17]
**Neurological disease**	Alzheimer's disease	Cognition	Tau, TSC1, TSC2, mTOR	Tau protein phosphorylation	Up	[3]
	Ischemic Stroke	Promote vascular repair	Plpp3, LPP3	mRNA Stabilization	Down	[8]
	Parkinson's Disease	Promote dopaminergic neuronal death	ATM, BNIP3	mRNA augmentation	Up	[7]
	Cognitive Dysfunction	Regulate neuronal oxidative stress	ATF3, HO-1, GCLM, γ-H2AX	mRNA augmentation	Down	[18]
	Neuralgia Oxidative Stress	Regulate oxidative stress	GPR177, WNT5a, TRPV1	mRNA Stabilization	Up	[19]
**Cardiovascular system diseases**	Myocardial infarction	Protect cardiomyocytes	IL-6, TNF-α, BCL2, Caspase-3	mRNA augmentation	Down	[20]
	Heart failure	regulate key genes	SERCA2a, MYH6/MYH7	mRNA augmentation	Down	[21]
	Atherosclerosis	Promote proliferation and migration	PPAR-γ, eNOS, KLF2	mRNA augmentation	Up	[22]

### 3.2 Bibliometric analysis of FTO and its functions

To understand the research landscape surrounding FTO, we analyzed 1,457 articles from the Web of Science database using VOSviewer. The bibliometric analysis focused on keyword co-occurrence to capture major research themes. Parameters included in this analysis were as follows: (1) Time frame: January 2021 - March 2025; (2) Primary keywords: “FTO”, “demethylase”, “methyltransferase”; (3) inclusion/exclusion criteria: The co-occurrence threshold is greater than or equal to 40 appearances; (4) The specific details of the clustering algorithm are as follows: 1)Type of analysis: co-occurrence, 2) Unit of analysis: all keywords, 3) Counting method: full counting; and (5) Data source: Web of Science Core Collection. The analysis revealed that FTO-related research is highly interconnected with gene regulation pathways, RNA-modifying enzymes, and RNA-binding proteins, consistent with a central role for FTO in the context of epitranscriptomic regulation. However, this approach is not without limitations. For example, for certain studies investigating FTO, some relevant studies may have been excluded due to failure to meet the keyword-based inclusion criteria.

**Figure 2.**
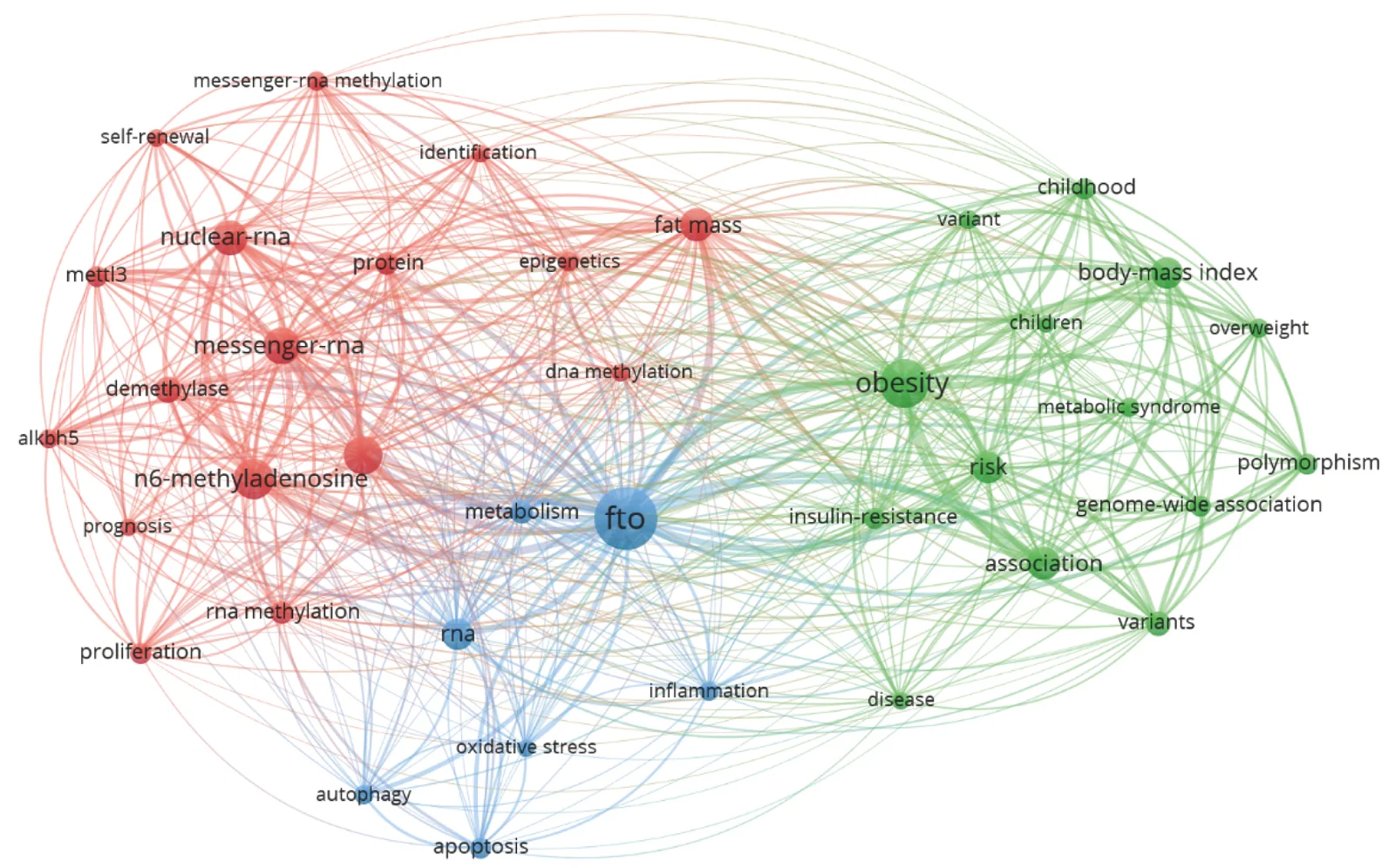
Visualization analysis of keywords related to FTO.

To construct a keyword co-occurrence map related to FTO, a minimum threshold of 40 times of keyword occurrence frequency was applied, resulting in the selection of 54 qualifying terms. From this pool, the top ten most frequently occurring keywords were identified as follows: "FTO" appeared 508 times; "obesity" 299 times; "N6-methyladenosine" 239 times; "methylation" 181 times; "messenger RNA" 180 times; "nuclear RNA" 153 times; "fat mass" 143 times; "FTO gene" 156 times; "risk" 127 times; and "body mass index" 123 times. These keywords indicate that current research hotspots predominantly center on involvement of FTO in RNA demethylation and its association with obesity-related risk factors (Fig. 2).

## 4. FTO in non-oncological diseases

### 4.1 Inflammation and fibrosis

Tissue injury and sustained inflammatory signaling are critical drivers of fibrogenesis [23]. Persistent inflammation often culminates in progressive fibrosis, ultimately leading to organ dysfunction and, in severe cases, death [24-26]. FTO acts as a regulatory modulator in both inflammatory and fibrotic pathways, which has significant implications for various non-malignant pathologies. For instance, in experimental models of silicosis, FTO expression is downregulated during early disease stages, resulting in elevated m6A methylation levels. This change exacerbates pulmonary inflammation and fibrotic remodeling, highlighting the promise of FTO as a potential molecular target for therapeutic intervention in silicosis [27]. Similarly, in models of renal fibrosis, which is a hallmark of chronic kidney disease, FTO silencing has been shown to reduce inflammation, cellular apoptosis, and impaired autophagic flux in TGF-β1-stimulated renal tubular epithelial cells (TECs) and in unilateral ureteral obstruction (UUO) models [28]. In a scleroderma mouse model, the overexpression of FTO reduces the tenascin C (TNC) mRNA levels by reducing the m6A level of this transcript, thereby alleviating skin fibrosis [29]. Overexpression of FTO can exacerbate HSC activation and liver fibrosis through autophagy [30]. FTO can also act as a cardioprotective factor by inhibiting post-infarction cardiac fibrosis through m6A modification [4]. FTO reportedly has no significant effect on corneal fibrosis [31]. As such, it appears that FTO may play distinct roles during the fibrotic processes associated with different diseases, ultimately facilitating the progression of renal fibrosis and liver fibrosis while slowing the progression of scleroderma and cardiac fibrosis. Moreover, FTO is expressed at a lower level and m6A levels are enhanced in the early stages of silicosis, which contributes to the exacerbation of pulmonary fibrosis.

### 4.2 Metabolic diseases

FTO is strongly involved in the pathogenesis of metabolic disorders such as obesity and diabetes, primarily through its regulation of RNA methylation and post-transcriptional gene expression [32]. Several studies have explored the role of FTO-mediated m6A demethylation in metabolic homeostasis [2,9].

#### 4.2.1 Mechanistic functions of FTO in obesity and metabolic regulation

FTO modulates the epitranscriptomic landscape of mRNAs involved in lipid metabolism, thereby influencing transcript maturation, stability, and translation to impact lipid metabolism and related disorders [17]. Genome-wide association studies (GWAS) have identified multiple single nucleotide polymorphisms (SNPs) in the first intron of the FTO gene that are strongly associated with obesity-related traits [33]. These associations have been validated across different ethnic populations [34]. Notably, the rs9939609 A allele is linked to higher BMI and increased obesity risk in Chinese cohorts, where carriers exhibit greater weight gain [35]. Another well-studied variant, rs1421085, predisposes individuals to obesity by disrupting regulatory elements that govern adipocyte differentiation [36]. These polymorphisms likely alter FTO gene regulatory elements or transcriptional control of FTO and neighboring genes, contributing to aberrant adipogenesis and energy imbalance.

Functionally, FTO influences several pathways that collectively contribute to obesity. In the central regulation of energy intake, FTO enhances the stability of the Ghrelin mRNA by limiting its m6A modification, leading to increased ghrelin secretion and appetite stimulation [37]. During adipogenesis, FTO promotes the formation of pro-adipogenic splicing variants of RUNX1T1 mRNA and upregulates cyclin A2/cyclin-dependent kinase 2 (CCNA2/CDK2) expression via a YTHDF2-dependent mechanism, thereby supporting preadipocyte proliferation [38-39]. In lipid homeostasis, FTO suppresses microRNAs such as miR-130 and miR-155, relieving their inhibitory effects on C/EBPβ and activating the PPAR-γ pathway, which enhances lipid droplet formation. Additionally, it stabilizes PLIN1 protein levels by inhibiting cathepsin B (CTSB)-mediated phosphorylation, thus reducing lipolytic activity. Furthermore, by modulating hypothalamic IRX3 expression and leptin signaling, FTO contributes to lipid accumulation through multiple synergistic mechanisms [17,40]. Similarly, in the liver, an increase in FTO levels and/or enhanced activity leads to an increase in glycogen synthesis, an increase in newly generated fat, and a decrease in fat breakdown and fatty acid oxidation [5]. FTO therefore exhibits a consistent net positive effect on fat formation and homeostasis.

FTO also plays a central role in glucose metabolism. Dysregulation of FTO alters the m6A landscape in pancreatic islet β-cells, leading to aberrant insulin secretion and signaling, thereby destabilizing homeostatic glucose balance. These disturbances create a mechanistic bridge linking obesity to type 2 diabetes [41].

#### 4.2.2 Advances in understanding the mechanistic regulation of lipid metabolism by FTO

FTO modulates lipid metabolism through specific epigenetic regulatory networks. Its Fe²^+^-dependent dioxygenase function facilitates dynamic demethylation of mRNAs involved in lipogenesis, fatty acid oxidation, and energy utilization. In hepatic tissue, FTO overexpression activates the SREBP1c pathway, enhancing the transcription of FAS and SCD, key enzymes in fatty acid biosynthesis. Simultaneously, it represses CPT-1, a critical enzyme in β-oxidation, promoting triglyceride accumulation and contributing to the development of non-alcoholic fatty liver disease [42]. In adipose tissue, FTO enhances lipid storage through three interrelated mechanisms: (1) facilitating alternative splicing of RUNX1T1 mRNA toward pro-adipogenic isoforms; (2) suppressing miR-130 to derepress PPAR-γ activity; and (3) stabilizing PLIN1 protein to augment lipid droplet retention [43-44]. Additionally, FTO disrupts lipolytic processes by inhibiting both IRX3 and leptin signaling, promoting a metabolic profile characterized by enhanced lipogenesis and reduced lipolysis [45]. In skeletal muscle, aberrant FTO expression downregulates oxidative pathways by suppressing PPARβ/δ and AMPK signaling, resulting in ectopic lipid deposition within myocytes [46-47]. In macrophages, FTO exerts bidirectional control over lipid handling by balancing CD36-mediated lipid uptake with AMPK-ABCA1-dependent cholesterol efflux, potentially modulating atherosclerotic plaque development [17]. Clinical studies have corroborated the association between FTO polymorphisms such as rs9939609 and obesity phenotypes across populations [48]. Tissue-specific FTO expression may also affect neuroendocrine pathways regulating appetite and energy expenditure, further reinforcing its candidacy as a therapeutic target for metabolic disease management.

**Figure 3.**
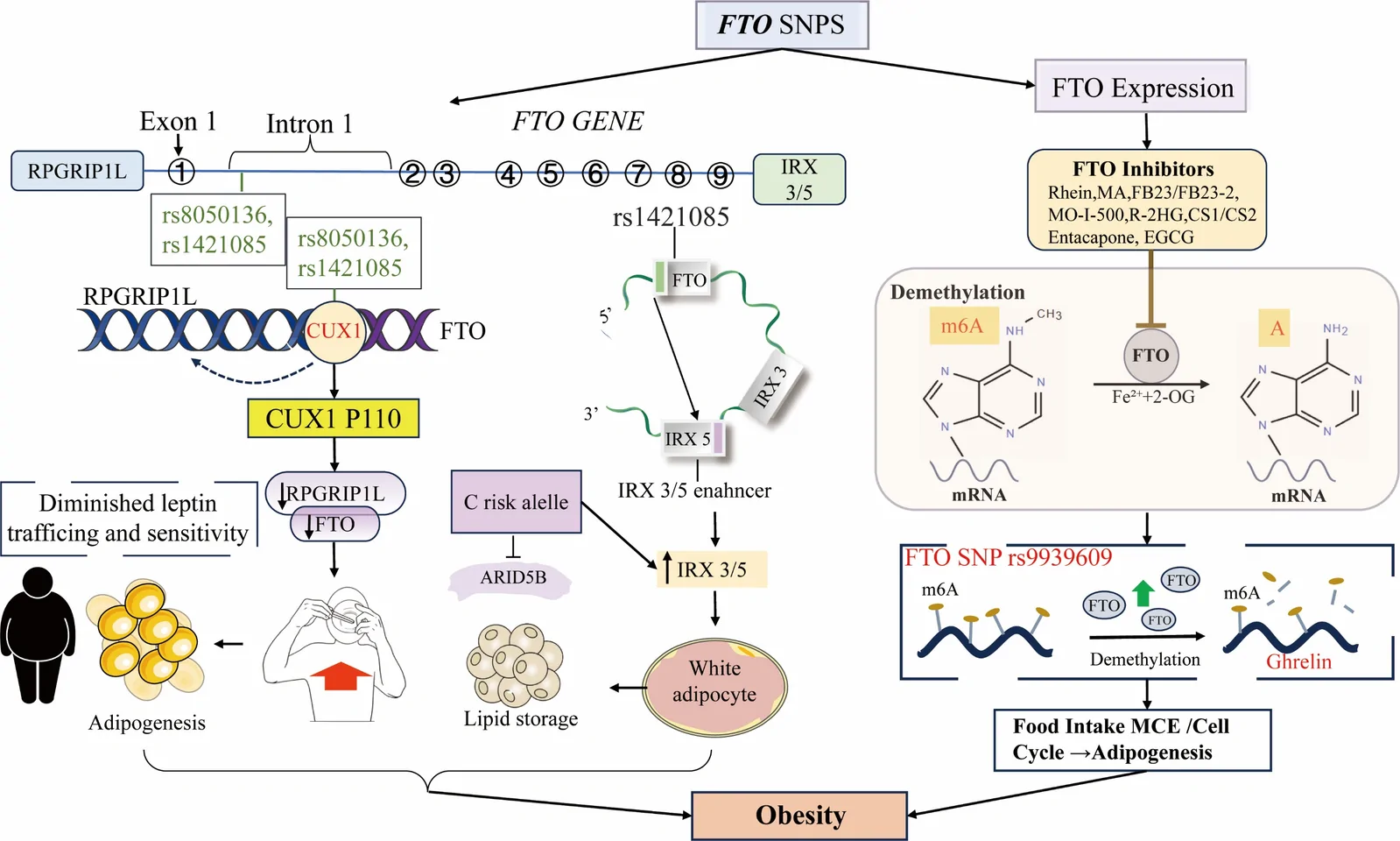
Molecular mechanisms of FTO SNPs in obesity.

The pathological significance of FTO gene polymorphisms in metabolic diseases is underscored by their interaction with other regulatory genes. Notably, the rs1421085 C allele and the rs8050136 A allele have been shown to reduce binding affinity for the cut-like homeobox 1 (CUX1) transcription factor. Specifically, the rs8050136 variant diminishes its interaction with the P110 isoform of CUX1, which is predominantly expressed in the hypothalamus. This reduced affinity is hypothesized to enhance the transcriptional activity of both FTO and RPGRIP1L (retinitis pigmentosa GTPase regulator-interacting protein 1-like). However, in the presence of the rs8050136 A allele, the regulatory impact of P110 on FTO and RPGRIP1L appears attenuated, thereby impairing leptin-mediated signaling and promoting adipocyte formation [49-50]. FTO polymorphisms exert their effects through interactions with other genes, such as IRX3 and IRX5, which are integral to adipogenesis and energy metabolism. In murine models, FTO variants have been implicated in modulating the browning of white adipose tissue, a process closely linked to thermogenesis and energy expenditure. The rs1421085 polymorphism, in particular, disrupts a conserved ARIDB5 (AT-rich interaction domain 5B) repressor binding motif, leading to overexpression of IRX3 and IRX5. These genes promote lipid storage by regulating the expression of downstream targets associated with lipid metabolism in white adipocytes, thereby contributing to the development of an obese phenotype [16, 50]. The involvement of FTO SNPs in obesity progression is further underscored by their participation in a highly complex molecular regulatory network. At the mechanistic level, FTO depends on Fe(II) and 2-oxoglutarate (2-OG) to catalyze the removal of m6A modifications from mRNA. This demethylation activity affects both the stability and translational efficiency of mRNA targets, thereby influencing various biological processes. Emerging evidence has identified several FTO inhibitors, such as rhein, gallic acid (GA), and epigallocatechin gallate (EGCG), which impede the demethylase function of FTO. By interfering with this enzymatic activity, these compounds may disrupt FTO-mediated pathways implicated in adipogenesis and offer promising avenues for therapeutic intervention in obesity and metabolic syndromes [51-53]. From the perspective of genetic regulation, FTO SNPs such as rs9939609 can drive enhanced FTO expression and promote the m6A demethylation of Ghrelin mRNA. This epigenetic modulation activates the ghrelin signaling pathway, enhancing appetite regulation, influencing cell cycle dynamics, and ultimately driving adipogenesis and lipid accumulation [8] (Fig. 3).

### 4.3 Nervous system diseases

In addition to its established role in metabolic disease, FTO has also been implicated in the pathophysiology of neurological conditions, including ischemic stroke, PD, and AD. Polymorphic variants of FTO may exacerbate cerebrovascular risk by disrupting lipid homeostasis, thereby predisposing individuals to ischemic events. In PD, elevated FTO expression has been linked to impaired microglial clearance of α-synuclein aggregates, intensifying neuroinflammation and neurodegeneration. Similarly, in AD, FTO appears to compromise neuronal energy metabolism and β-amyloid regulation, contributing to cognitive impairment [6,54-55].

#### 4.3.1 FTO regulatory activity and therapeutic potential in Alzheimer's disease

Recent studies using animal models of AD have established FTO as a key regulatory factor in disease progression. Elevated FTO expression has been observed in obese and diabetic models, as well as in the triple-transgenic AD (3xTg-AD) mouse model (APPswe, PS1M146V, and Tau P301L). Notably, both mRNA and protein levels of FTO are significantly higher in the brains of 3xTg-AD mice compared to wild-type counterparts, suggesting that FTO contributes to AD via epigenetic mechanisms. One central pathway involves the impairment of insulin signaling, which upregulates FTO and amplifies Tau protein hyperphosphorylation. Mechanistically, FTO regulates the TSC1-mTOR-Tau axis: increased FTO expression downregulates Tsc1 mRNA, thereby relieving inhibition on the mTOR pathway, which in turn becomes hyperactivated, resulting in excessive Tau phosphorylation [3,56]. When FTO causes the accumulation of Tau phosphorylation, it drives the cognitive decline and disease progression that characterize AD. Experimental silencing of FTO has been shown to reduce phosphorylated Tau accumulation in neurons, an effect that can be reversed with the mTOR inhibitor rapamycin, thus confirming the pathway’s involvement [57]. Importantly, FTO does not appear to significantly influence β-amyloid deposition, but rather exerts its pathological effects primarily through Tau-related mechanisms. Neuron-specific FTO knockout in AD models has been demonstrated to restore spatial learning and memory performance, highlighting its role in cognitive dysfunction [3]. These findings underscore the therapeutic potential of targeting the FTO-mTOR axis in AD. Ongoing research is exploring FTO-specific inhibitors, gene-editing strategies, and combination therapies aimed at modulating Tau post-translational modifications to achieve precision treatment of AD. However, in AD model mice and HT22 hippocampal neuronal cells, the reduction of FTO expression may promote the pathogenesis of AD by activating the Notch1 pathway and interfering with the immune response [58]. A prospective population-based study of 1,003 persons without dementia has shown that the interaction between the FTO AA genotype and the APOE ϵ4 genotype increases the risk of developing AD [59].

#### 4.3.2 FTO regulatory activity and therapeutic potential in Parkinson's disease

In PD, aberrant FTO expression has been shown to drive disease progression. Elevated levels of FTO mRNA and protein have been detected in both 1-methyl-4-phenyl-1,2,3,6-tetrahydropyridine (MPTP)-induced mouse models and 1-methyl-4-phenylpyridinium (MPP^+^)-treated dopaminergic neurons. Clinical samples further confirm increased FTO expression in the striatal regions of PD patients relative to healthy individuals. FTO promotes the m6A demethylation of BAP1, leading to an increase in BAP1 expression levels. BAP1, in turn, interacts with p53 to reduce SLC7A11 levels and promote neuronal ferroptosis [60]. Therefore, to alleviate the abnormal expression of FTO and symptoms, precision interventions can be deployed targeting FTO in the striatum of PD patients to efficiently treat this disease.

Experimental suppression of FTO reduces the expression of α-synuclein and neuronal apoptosis, while restoring levels of tyrosine hydroxylase (TH), a key enzyme in dopamine synthesis. This neuroprotective effect appears to be mediated through the stabilization of the mRNA encoding the expression of cellular death-related factor ataxia telangiectasia mutated (ATM) via m6A-dependent pathways, thereby alleviating the expression of this pro-apoptotic protein and mitigating damage to neurons [6]. Inhibiting the expression of FTO is beneficial for PD. As such, efforts to suppress FTO expression may provide an effective means of alleviating the symptoms of PD and improving nervous system functionality. Additionally, FTO may influence dopamine neurotransmission by regulating genes such as GRIN1, which encodes a subunit of the NMDA receptor, although the precise molecular circuitry remains to be fully elucidated [61]. Capitalizing on these findings, researchers have developed a novel siRNA delivery system (Exo-siFTO) using mesenchymal stem cell-derived exosomes encapsulating siRNAs of interest. This system is capable of crossing the blood-brain barrier and selectively targeting FTO expression within the striatum. In preclinical models, Exo-siFTO treatment effectively reduces dopaminergic neuron loss and significantly improves motor function, offering a promising therapeutic strategy for PD [6]. In PD model systems, FTO expression tends to be elevated. Therefore, FTO inhibitors may have a significant effect in this experimental setting, alleviating patient PD symptoms and even achieving therapeutic results.

#### 4.3.3 Mechanistic role of FTO in post-stroke vascular repair and neurological recovery

In the aftermath of ischemic stroke, a disruption in m6A RNA methylation patterns within brain tissues has been closely associated with a decline in FTO protein levels. Experimental data indicate that the demethylase activity of FTO is significantly reduced in the peri-infarct cortical region. Notably, the circular RNA circSCMH1 has been identified as a critical modulator that interacts with FTO to enhance its nuclear localization and stimulate the m6A demethylation of phospholipid phosphatase 3 (Plpp3) mRNA. This interaction facilitates the stabilization of Plpp3 transcripts, thereby promoting vascular remodeling [7]. Validation of this mechanism in both photothrombosis models and non-human primate stroke models revealed that delivery of extracellular vesicles enriched with circSCMH1 increased vascular density within infarcted areas by 30% to 50%. In parallel, gene-edited mice engineered for endothelial cell-specific overexpression of FTO exhibited markedly accelerated vascular regeneration and improved functional recovery, thereby underscoring the central role of the FTO-Plpp3 signaling axis in post-stroke angiogenesis [62]. In addition to the above-mentioned protective effect of FTO on the nerves after stroke, there are also studies that have proved that FTO inhibits ERS-mediated cell apoptosis by regulating the miR-503-5p/USP10 axis, and has clarified that FTO has a neuroprotective effect on acute ischemic stroke (AIS) [63]. Another study has shown that the better the prognosis of patients with AIS, the higher the expression level of FTO. And through a cohort study involving 201 patients and 53 healthy controls, it was proved that an elevated plasma FTO level can predict a favorable outcome after AIS [64].

Beyond enhancing perfusion in damaged regions, improved vascular integrity establishes a supportive neurotrophic environment that facilitates neuronal repair. Behavioral assays demonstrated that mice overexpressing FTO exhibited enhanced motor recovery, including a 40% reduction in foot fault errors and a 25% improvement in adhesive removal response times across different stroke paradigms. These functional gains may be attributed to increased levels of neurotrophic factors, such as brain-derived neurotrophic factor (BDNF) and vascular endothelial growth factor (VEGF), transported through newly formed vasculature [65]. Although FTO does not directly modulate neuronal signaling, its influence on the vascular microenvironment plays a critical indirect role in promoting neural regeneration. These findings offer a novel therapeutic avenue for targeted stroke recovery.

**Figure 4.**
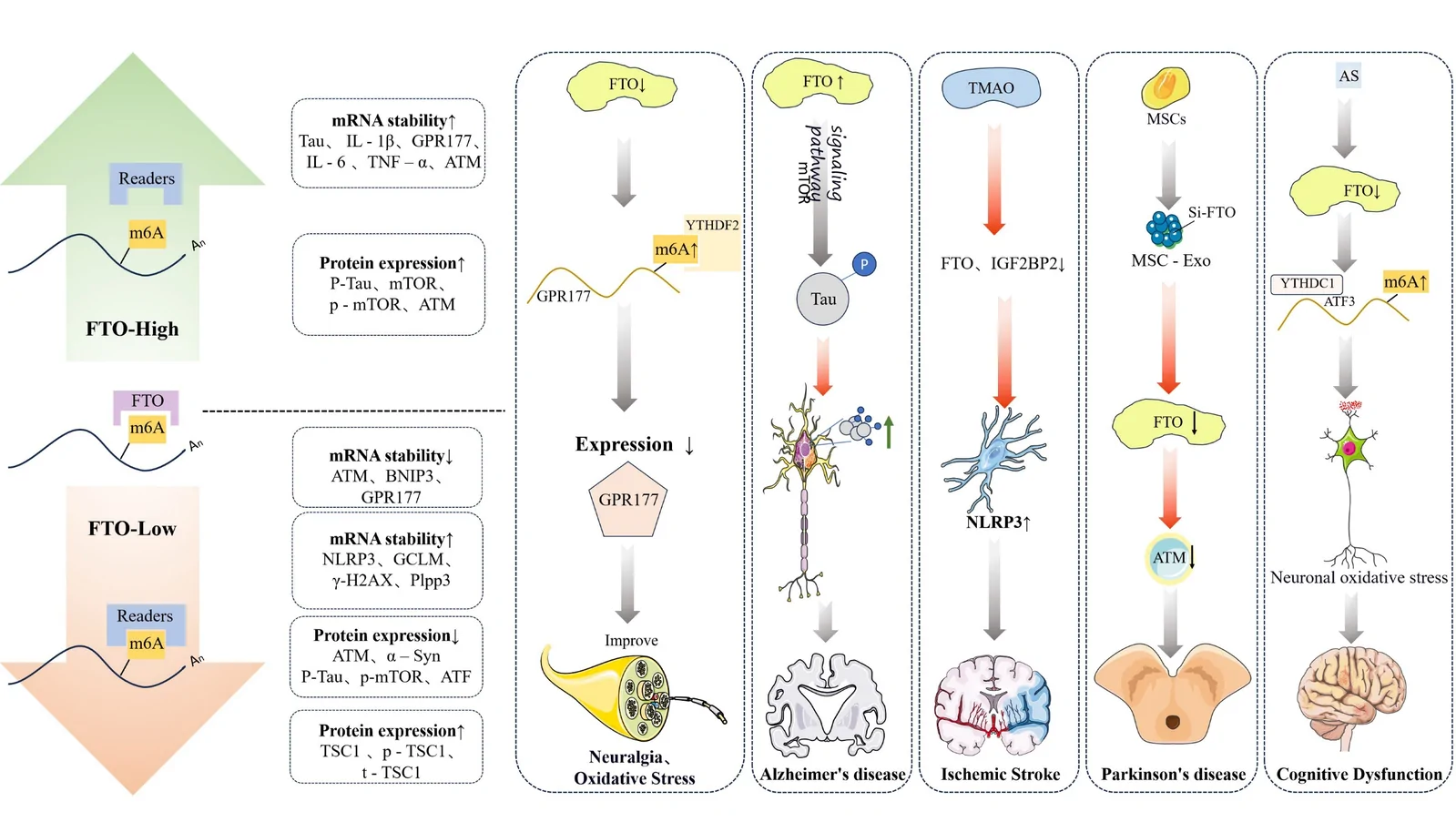
Molecular mechanisms of FTO in neurological disorders.

At a mechanistic level, FTO modulates disease outcomes by altering mRNA stability and subsequent protein expression. Upregulation of FTO increases demethylation activity, leading to decreased m6A modifications and reduced binding of m6A reader proteins. This change enhances the stability and expression of mRNAs encoding key pathological proteins, such as Tau, IL-1β, IL-6, GPR177, TNF-α, and ATM, culminating in elevated protein levels of p-Tau, mTOR, and TSC1 [66-67]. Conversely, downregulation of FTO leads to elevated m6A methylation and increased reader recognition, stabilizing mRNAs such as BNIP3, GCLM, γ-H2AX, and Plpp3. This modulation results in reduced expression of ATM, α-synuclein, p-Tau, and p-mTOR, while boosting levels of total and phosphorylated TSC1 [3,6]. FTO also engages diverse molecular pathways across multiple neurological contexts. In neuropathic pain and oxidative stress, reduced FTO expression enhances m6A methylation on GPR177 mRNA, promoting its degradation and thereby alleviating pain and oxidative damage [19]. In AD, elevated FTO expression drives Tau hyperphosphorylation via mTOR signaling, exacerbating neurodegenerative progression. In ischemic stroke, TMAO-mediated modulation of FTO and its interaction with IGF2BP2 activates NLRP3 inflammasome expression, further contributing to neuroinflammation [54]. In PD, downregulated FTO expression in mesenchymal stem cell (MSC)-derived exosomes impairs ATM signaling and aggravates dopaminergic neuron loss in the substantia nigra. Additionally, environmental toxins such as arsenic suppress FTO levels, increasing m6A modification on ATF3 mRNA. These marks are recognized by YTHDC1, which downregulates ATF3, impairs antioxidant defense mechanisms, and induces neuronal oxidative stress and cognitive dysfunction [6] (Fig. 4).

### 4.4 Cardiovascular diseases

Cardiovascular diseases (CVDs) remain a leading global cause of death, with atherosclerosis serving as a foundational pathological condition characterized by lipid accumulation and chronic inflammation in arterial walls. Rupture of atherosclerotic plaques may precipitate acute myocardial infarction (MI), frequently progressing to heart failure (HF) in survivors. This trajectory highlights the interconnected nature of atherosclerosis, MI, and HF [68]. Recent research has shown that FTO plays a multifaceted role in CVD pathogenesis. FTO dynamically regulates gene expression in lipid metabolism, inflammation, vascular remodeling, and cardiomyocyte biology through reversible m6A-dependent RNA modifications [69]. The sections below detail recent findings focused on the molecular and cellular effects of FTO in various cardiovascular conditions.

#### 4.4.1 Atherosclerosis

Research has increasingly focused on the regulatory impact of FTO in the development of atherosclerosis. FTO appears to influence lipid homeostasis by modulating cholesterol synthesis and absorption pathways. Carriers of FTO risk alleles display elevated activity of key cholesterol biosynthesis enzymes, such as 3-hydroxy-3-methylglutaryl-coenzyme A (HMG-CoA) reductase and increased intestinal absorption via Niemann-Pick C1-like 1 (NPC1L1), resulting in higher plasma levels of total cholesterol and LDL-C [69-70]. These changes impair ATP-binding cassette transporter A1 (ABCA1)-mediated reverse cholesterol transport and exacerbate lipid deposition in vessel walls. FTO also exerts a biphasic influence on fatty acid metabolism. At physiological expression levels, FTO activates peroxisome proliferator-activated receptor-alpha (PPAR-α), enhancing fatty acid β-oxidation and reducing lipid accumulation [71]. However, FTO overexpression disrupts redox homeostasis by increasing oxidative stress markers like malondialdehyde and promoting a pro-inflammatory microenvironment.

FTO contributes to macrophage polarization and inflammation through m6A-dependent mechanisms. Stabilization of suppressor of cytokine signaling 3 (SOCS3) mRNA by FTO reduces inhibition of the JAK-STAT pathway, driving M1 macrophage activation [72]. This transition elevates inflammatory cytokines such as IL-6 and TNF-α and upregulates CD36, thereby promoting foam cell formation via enhanced lipid uptake. Additionally, FTO-mediated oxidative stress is linked to mitochondrial dysfunction. Specifically, it downregulates mitochondrial biogenesis through suppression of peroxisome proliferator-activated receptor gamma coactivator 1-alpha (PGC-1α), while concurrently activating the NOX4/NADPH oxidase complex, resulting in excessive mitochondrial superoxide generation. This oxidative imbalance impairs endothelial nitric oxide synthase (eNOS) function and facilitates oxidation of LDL-C to ox-LDL, intensifying vascular inflammation and plaque formation [73-74].

FTO also orchestrates complex signaling cascades regulating vascular smooth muscle cell (VSMC) plasticity, a critical event in plaque development. Through stabilization of Kruppel-like factor 4 (KLF4) mRNA, FTO promotes the phenotypic switch of vascular smooth muscle cells (VSMCs) from contractile to synthetic forms. This is evidenced by decreased expression of contractile markers (e.g., SM22α) and increased expression of synthetic markers such as osteopontin (OPN) and matrix metalloproteinase-9 (MMP-9), leading to extracellular matrix (ECM) remodeling characterized by collagen and fibronectin deposition. Moreover, FTO enhances VSMC proliferation by upregulating cyclin D1 expression, which increases S-phase cell entry by approximately 30%, as confirmed via EdU incorporation assays. Notably, the effects of FTO vary with plaque maturity. During early plaque formation, it promotes VSMC migration through the CXCR4/CXCL12 axis, whereas in advanced plaques, it downregulates MMP-2, impairing ECM degradation. This dual role paradoxically increases the mechanical fragility of the fibrous cap, contributing to plaque rupture risk despite increased cap thickness [75-77]. Collectively, FTO-mediated pathways constitute an intricate molecular framework driving atherosclerotic progression. These findings position FTO as a promising therapeutic target for the prevention and treatment of CVDs.

#### 4.4.2 Myocardial infarction

Studies published in recent years have suggested a role for FTO dysregulation in the pathogenesis of MI, HF, and atherosclerosis via multifaceted molecular pathways. FTO influences systemic energy balance in part through central neural regulation, and individuals carrying risk alleles frequently exhibit dietary preferences oriented toward high-fat and high-sugar foods, promoting obesity-related dyslipidemia. This metabolic imbalance contributes to endothelial dysfunction characterized by impaired nitric oxide generation and elevated oxidative stress, which accelerates atherosclerotic plaque development. Experimental models further show that FTO deficiency or malfunction reduces adipocyte maturation and disrupts cholesterol metabolism, leading to increased circulating triglycerides and LDL, with these factors being strongly correlated with endothelial injury [78-79].

At the level of inflammation and oxidative stress, FTO upregulates pro-inflammatory mediators such as IL-6 and TNF-α while suppressing antioxidant enzymes like superoxide dismutase. These shifts create a harmful cycle of inflammation and reactive oxygen species accumulation, driving cardiomyocyte apoptosis through altered Bcl-2 family protein ratios and enhanced caspase 3 activation. The resultant loss of myocytes undermines systolic function [20,79-80]. Additionally, FTO promotes pathological hypertrophy by activating PI3K/AKT signaling, which results in cardiomyocyte enlargement with reduced contractile capacity, providing a structural foundation for subsequent HF onset [81-82].

FTO also contributes to vascular remodeling by triggering phenotypic switching in VSMCs, which is closely involved in MI incidence [72]. The overexpression of FTO facilitates VSMC switching from a contractile to a synthetic phenotype, from a contractile to a synthetic state. This transition is characterized by upregulation of proliferation markers such as PCNA and Ki-67 and downregulation of migratory gene expression, leading to vascular wall thickening, extracellular matrix deposition, and increased plaque burden [72,83]. Additionally, FTO activation triggers TGF-β/Smad signaling, enhancing collagen deposition within the myocardium, with this being a hallmark of fibrotic remodeling in HF [84-86]. Collectively, these findings reinforce the role of FTO at the intersection of metabolic dysfunction, inflammation, and vascular pathology, supporting its potential as a novel target in CVD therapy.

**Figure 5.**
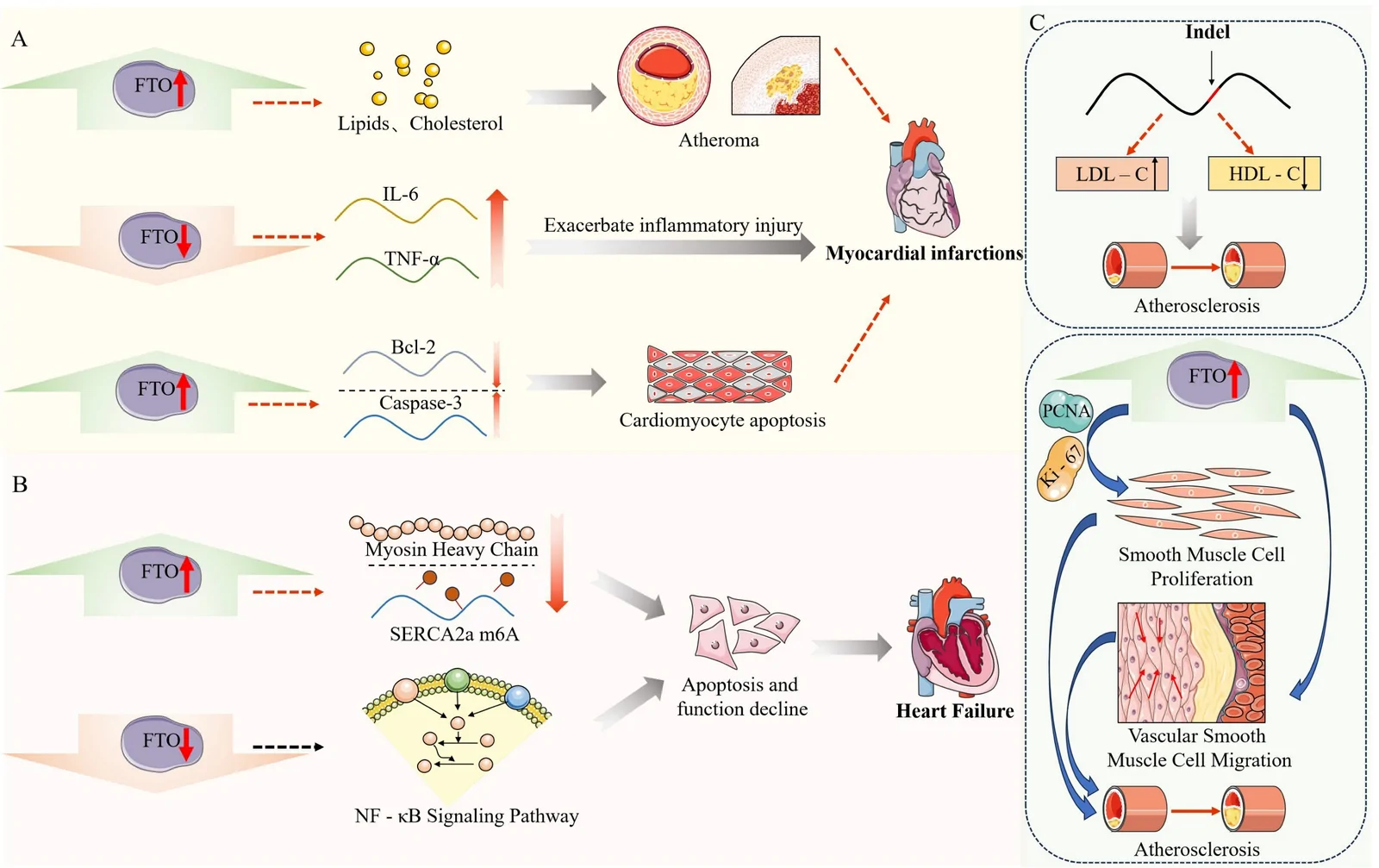
**Molecular mechanisms of FTO in cardiovascular diseases**. (**A**) FTO affects the survival of cardiac muscle cells and the recovery of cardiac function, thereby accelerating the progression of myocardial infarction; (B) FTO causes cell apoptosis, thereby promoting the occurrence of heart failure; (C) FTO reduces the expression of HDL, and increases the expression of LDL, PCNA and Ki-67, promoting the occurrence and development of atherosclerosis.

#### 4.4.3 Heart failure

FTO drives multiple maladaptive processes in HF. While conventional therapies (e.g., ACE inhibitors, β-blockers) alleviate symptoms and improve outcomes [87], FTO-mediated pathways perpetuate HF progression. Specifically, FTO enhances angiotensin II production, activating the AT1R/NF κB axis, which promotes cardiomyocyte hypertrophy, especially under conditions of β1-adrenergic receptor overexpression and dysregulated cAMP/PKA signaling observed in sympathetic overactivity [88-90]. These events culminate in cellular injury and functional decline. Through epigenetic regulation, FTO also alters extracellular matrix (ECM) dynamics by modulating collagen synthesis and degradation. FTO enhances the m6A methylation of the COL1A1 and COL3A1 mRNAs while downregulating MMP-2 expression, leading to dysregulated collagen accumulation and scarring [91-92]. This remodeling impairs contractile protein expression and reduces m6A modifications on SERCA2a mRNA, thereby enhancing its translation to sustain cardiac energy metabolism [93-94]. In contrast, FTO silencing increases m6A methylation of TLR4, promoting YTHDF1 binding and NF-κB activation (phosphorylated p65), which in turn triggers cardiomyocyte apoptosis, pyroptosis, and oxidative stress, thereby further accelerating HF [95].

**Figure 6.**
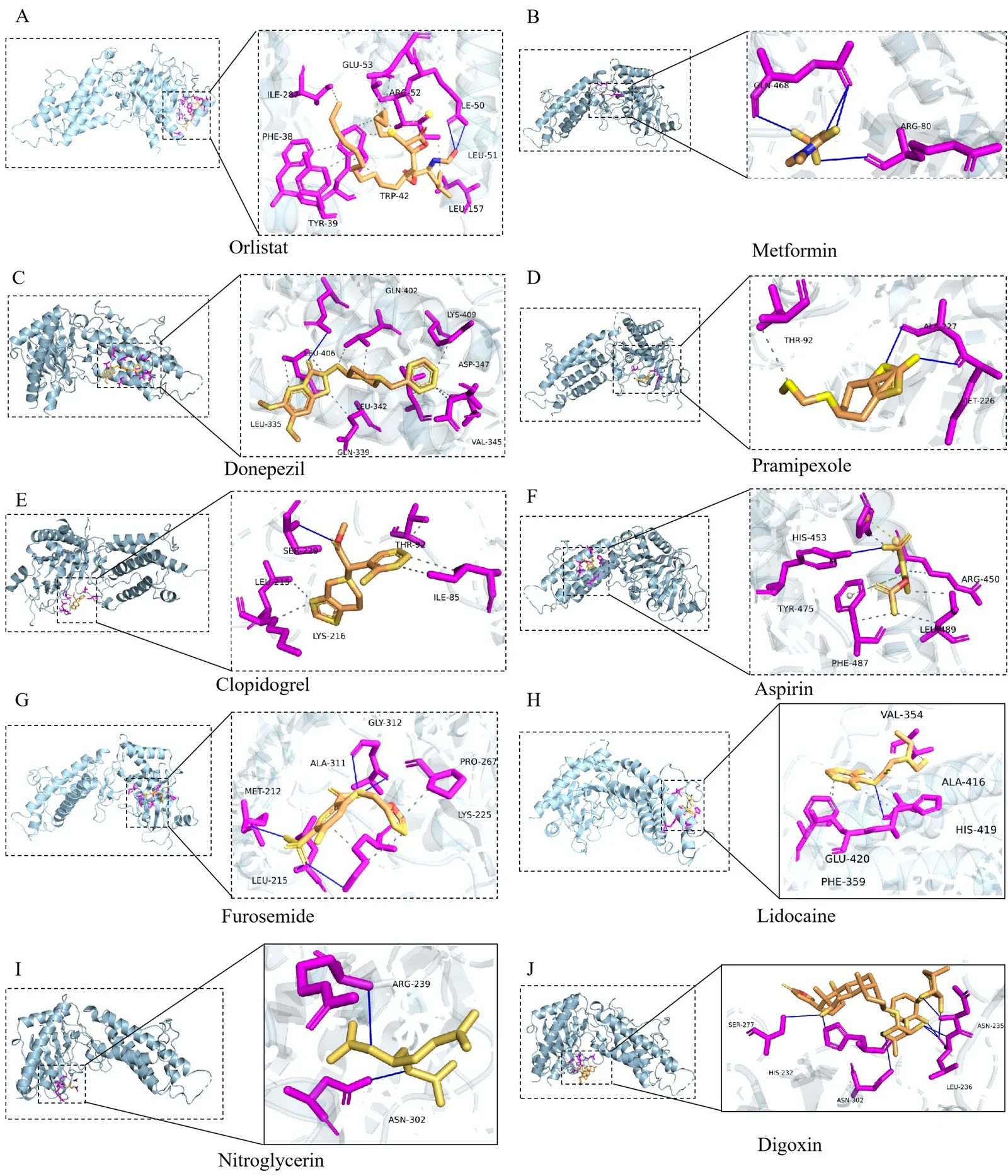
**Docking interactions between FTO and conventional therapeutic drugs**. (**A**) Docking of Orlistat and FTO; (B) Docking of Metformin and FTO; (C) Docking of Donepezil and FTO; (D) Docking of Pramipexole and FTO; (E) Docking of Clopidogrel and FTO; (F) Docking of Aspirin and FTO; (G) Docking of Furosemide and FTO; (H) Docking of Lidocaine and FTO; (I) Docking of Nitroglycerin and FTO; (J) Docking of Digoxin and FTO.

FTO has emerged as a pivotal regulator in the development and progression of CVD through its involvement in multiple molecular pathways. In cardiomyocytes, FTO influences inflammatory responses by modulating the expression of pro-inflammatory cytokines such as interleukin-6 (IL-6) and tumor necrosis factor-alpha (TNF-α), thereby impacting cell viability and cardiac recovery following injury. Aberrant FTO expression also impairs lipid metabolism, leading to the pathological accumulation of triglycerides and cholesterol within cardiac tissues. Furthermore, FTO regulates apoptosis via modulation of Bcl-2 family proteins and activation of caspases, processes that are directly linked to the severity and outcome of MI (Fig. 5A). On a functional level, FTO contributes to reduced expression of contractile proteins, including myosin heavy chain and actin, while simultaneously promoting HF by activating the NF-κB signaling cascade (Fig. 5B). In the vascular system, mutations or dysregulation of FTO are associated with increased low-density LDL-C levels and a concomitant reduction in high-density lipoprotein cholesterol (HDL-C). Additionally, FTO overexpression has been shown to enhance VSMC proliferation, as evidenced by upregulation of proliferation markers such as proliferating cell nuclear antigen (PCNA) and Ki-67. Simultaneously, FTO suppresses the expression of genes involved in VSMC migration, thereby contributing to the pathophysiology of atherosclerosis (Fig. 5C).

These molecular interactions underscore the central role of FTO in the complex regulatory network that drives HF. Interestingly, in the early compensatory phase of HF, FTO may transiently support cardiac function by upregulating pathways associated with energy metabolism. As the disease progresses into the decompensated phase, underlying pathological conditions such as atherosclerosis and MI can lead to amplified disease severity, culminating in clinical deterioration and impaired quality of life. Future studies should focus on delineating the spatial and temporal specificity of FTO activity across different stages of cardiovascular pathology and within distinct anatomical regions. Such research will be essential for establishing a mechanistic framework that can inform the development of targeted therapies aimed at modulating FTO function in cardiovascular disease.

## 5. Opportunities and challenges associated with FTO

Recent studies have highlighted the prominent role of the FTO in various malignancies. However, it is increasingly evident that FTO also influences a range of non-cancerous conditions that significantly affect public health and quality of life. Given its involvement in diverse pathological processes, FTO has emerged as a promising therapeutic target not only for cancer but also for metabolic, cardiovascular, and neurodegenerative diseases. To investigate whether commonly used drugs for non-oncologic conditions might interact with FTO, we employed molecular docking techniques using AutoDockTools 1.5.7 software. This analysis aimed to evaluate the binding potential between FTO and a selection of conventional drugs widely used in the management of non-cancerous diseases. The docking setup included specific parameters, namely the dimensions of the docking grid and the same coordinates of the docking center at x = 21.82, y = -12.39, and z = -28.47. Ligand structures were sourced from the PubChem database (https://pubchem.ncbi.nlm.nih.gov/). For docking, three-dimensional conformations were either directly downloaded or, when only two-dimensional representations were available, converted into 3D structures using ChemDraw. The docking results of FTO with the classical therapeutic drugs are shown in Figure 6, Table 2, and Figure 7.

**Table 2:** Results of docking between FTO and compounds.

Diseases	Abbreviation of diseases	Classic drugs	Binding energy
**Obesity**	Obesity	Orlistat	-5.6
**Type 2 Diabetes Mellitus**	T2DM	Metformin	-5.5
**Alzheimer's Disease**	AD	Donepezil	-7.2
**Parkinson’s Disease**	PD	Pramipexole	-4.7
**Atherosclerotic Cardiovascular Disease**	ACD	Clopidogrel	-5.9
**Ischemic Stroke**	IS	Aspirin	-5.3
**Congestive Heart Failure**	CHF	Furosemide	-6.1
**Ventricular Arrhythmias**	VA	Lidocaine	-5.3
**Angina Pectoris**	AP	Nitroglycerin	-4.9
**Heart Failure**	HF	Digoxin	-7.5

*FTO and Orlistat:* Docking analysis revealed that Orlistat binds to FTO with an affinity of -5.6 kcal/mol. The interaction profile includes 13 hydrophobic contacts involving the Phe38, Ile287, Glu53, Arg52, Tyr39, Trp43, Ile50, Leu51, and Leu157 residues. Additionally, Arg52 participates in two hydrogen bonds and one salt bridge, confirming a stable interaction (Fig. 6A). The dimensions of the docking box were size_x = 76.95, size_y = 68.85, and size_z = 70.2.

*FTO and Metformin:* Metformin exhibited a docking score of -5.5 kcal/mol when bound to FTO. The interaction is mediated by four hydrogen bonds involving Gln468 and Arg80. Although the binding energy suggests a moderate affinity, the presence of stable polar interactions supports the plausibility of complex formation (Fig. 6B). The docking box measured size_x = 75.95, size_y = 57.87, and size_z = 71.13.

*FTO and Donepezil:* Donepezil exhibited a binding affinity of -7.2 kcal/mol with FTO. The complex involves 11 hydrophobic bonds including Leu335, Leu406, Lys409, Asp347, Val345, Gln339, and Leu342, along with a hydrogen bond formed with Gln402 (Fig. 6C). The docking grid dimensions were size_x = 93.90, size_y = 66.08, and size_z = 85.21.

*FTO and Pramipexole:* Pramipexole exhibited a binding energy of -4.7 kcal/mol when interacting with FTO, with a single hydrophobic contact with Thr92 and two hydrogen bonds involving Met226 and Ala227 (Fig. 6D). The size of the docking box was size_x = 74.36, size_y = 59.75, and size_z = 70.37.

*FTO and Clopidogrel:* Clopidogrel exhibited a binding affinity of -5.9 kcal/mol with FTO. The interaction included contacts with the hydrophobic Ile85, Thr92, Leu215, and Lys216 residues, as well as a hydrogen bond formed with Ser229 (Fig. 6E). The size of the docking box was the same as that for the docking of FTO and Pramipexole.

*FTO and Aspirin:* Aspirin was found to interact with FTO with a docking score of -5.3 kcal/mol. The binding involved five hydrophobic bonds (Arg450, His453, Phe487, and Leu489) as well as a hydrogen bond with Tyr475. Furthermore, Phe487 contributed to a π-stacking interaction, and His453 formed a salt bridge (Fig. 6F). The size of the docking box was size_x = 93.1, size_y = 93.1, and size_z = 73.89.

*FTO and Furosemide:* Furosemide demonstrated a binding affinity of -6.1 kcal/mol, interacting with FTO through three hydrophobic bonds (Lys225, Pro267) and five hydrogen bonds formed with Met212, Leu215, Lys225, Ala311, and Gly312 (Fig. 6G). The size of the docking box was the same as that for the docking of FTO and Pramipexole.

*FTO and Lidocaine:* Molecular docking analysis indicated that lidocaine binds to FTO with a binding energy of -5.3 kcal/mol. This interaction is primarily stabilized by five hydrophobic contacts involving Val354, Ala416, His419, Glu420, and Phe359, along with one hydrogen bond formed with Ala416 (Fig. 6H). The size of the docking box was the same as that for the docking of FTO and Pramipexole.

*FTO and Nitroglycerin:* Nitroglycerin exhibited a binding affinity of -4.9 kcal/mol with FTO, forming two hydrogen bonds involving Arg239 and Asn302 (Fig. 6I). The size of the docking box was the same as that for the docking of FTO and Pramipexole.

*FTO and Digoxin:* Digoxin demonstrated one of the strongest interactions with FTO, with a binding energy of -7.5 kcal/mol supported by eight hydrogen bonds involving the His232, Leu236, Asn235, Ser277, and Asn302 residues (Fig. 6). The size of the docking box was the same as that for the docking of FTO and Pramipexole.

For reference, compounds derived from *Annona muricata* have shown favorable FTO binding profiles, with binding energies ranging from -5.6 to -9.2 kcal/mol [96]. The binding affinities of the non-oncologic drugs analyzed in this study range between -4.7 and -7.5 kcal/mol when interacting with FTO. The majority of the complexes, including those formed between FTO and Orlistat, Metformin, Pramipexole, Clopidogrel, Aspirin, Furosemide, Lidocaine, and Nitroglycerin, displayed moderate binding affinities, ranging from -5 to -7 kcal/mol. In contrast, a limited number of complexes, specifically those formed between FTO and both Donepezil and Digoxin, exhibited stronger interactions, with binding energies less than -7 kcal/mol. Only two compounds (Pramipexole and Nitroglycerin) showed binding energies less negative than -5 kcal/mol (-4.7 and -4.9 kcal/mol, respectively). Collectively, these results support the hypothesis that the selected drugs form stable complexes with FTO. Further investigation is required to elucidate FTO’s molecular roles across various pathologies and to harness its therapeutic potential through targeted modulation.

FTO has therapeutic potential in metabolic diseases, neurological disorders, and CVDs, but it is also subject to certain limitations. In metabolic diseases, it is worth noting that inhibiting FTO may lead to metabolic disorders and even a decrease in metabolic rate. In neurological disorders, the safety of central nervous system delivery strategies needs to be ensured, appropriately tailoring immunogenicity and tissue specificity to prevent interference with the normal physiological functions of FTO. In CVDs, FTO exhibits opposing effects during different stages of HF, which further increases the difficulty of therapeutic intervention.

## 6. Discussion

FTO inhibitors are generally classified into three categories based on their mechanism of action. The first includes iron ion chelators [97]. The second comprises competitive inhibitors of 2-oxoglutarate (2-OG), such as R-2-hydroxyglutarate (R-2HG) and methoxyfenic acid (MA). R-2-hydroxyglutarate (R-2HG) inhibits the activity of FTO, thereby reducing the proliferation of cancer cells and inhibiting aerobic glycolysis [98]. Methoxyfenic acid (MA) and its derivative MA2 can block the activity of FTO and down-regulate the expression of MYC in glioblastoma. The third category includes substrate-competitive inhibitors such as FTO-43N, which modulate the Wnt/PI3K-Akt signaling cascade, particularly in gastric cancer. Each inhibitor type displays unique pharmacodynamic profiles and mechanisms of action [99]. Despite encouraging preclinical results, existing FTO inhibitors face limitations regarding clinical applicability due to suboptimal target selectivity and pharmacokinetic profiles. Since lipid metabolism is closely tied to cancer pathogenesis and FTO is a central regulator of lipid homeostasis, FTO-targeting compounds may preferentially accumulate in tumor cells. Nevertheless, enhancing target specificity remains essential to reduce off-target effects and improve clinical efficacy [99]. Epidemiological studies also underscore FTO's relevance in non-cancerous conditions. Cohort analyses have linked FTO polymorphisms to increased BMI in pediatric populations. Additional evidence suggests that FTO expression is negatively correlated with the incidence of intrauterine growth restriction (IUGR) [100]. These findings emphasize the translational value of FTO-related biomarkers in clinical diagnostics, therapeutic planning, and prognostic evaluation.

Molecular studies have uncovered a negative correlation between FTO and METTL3 expression at both the mRNA and DNA methylation levels in clear cell renal cell carcinoma (CCRCC) [101]. In colorectal cancer (CRC), reduced expression of FTO and ALKBH5 activates the FOXO signaling pathway via m6A RNA methylation. This downregulation also leads to elevated METTL3 expression and suppressed METTL14 expression, thereby facilitating tumor progression [102].

A conserved FTO-TSC1-mTOR signaling axis has been identified across diverse pathological contexts. Diminished TSC1 expression within this axis contributes to AD pathophysiology by increasing Tau phosphorylation [3,62]. Similarly, TSC1 deficiency promotes lipid biosynthesis by upregulating acetyl-CoA carboxylase 1 (ACC1), thereby playing a pathogenic role in metabolic disorders [103]. Furthermore, TSC1-mediated regulation of mTOR signaling in endothelial cells is critical for normal vascular development and embryogenesis [104].

FTO serves as a hub linking metabolic and epigenetic pathways and may exert protective or pathological effects depending on the disease context and stage. While FTO overexpression is associated with the progression of several diseases, including an elevated risk of atherosclerotic stroke, it can also play neuroprotective roles, such as enhancing recovery after ischemic injury [7].

In the silicosis model, the downregulation of FTO expression leads to an increase in m6A methylation levels, exacerbating pulmonary inflammation and fibrotic remodeling. Interestingly, in HSCs, a reduction in FTO results in the downregulation of autophagy-related 9A (ATG9A) at the mRNA level. This, in turn, may exert an anti-fibrotic effect through the phosphoinositide 3-kinase/protein kinase B/mechanistic target of rapamycin (PI3K/AKT/mTOR) and TGFβ1/Smad3 pathways [105].

FTO not only participates in the pathogenesis of obesity and metabolic diseases, but also plays a potentially crucial role in carcinogenesis through different signaling pathways, including m6A demethylation. Furthermore, it has been reported that the role of FTO in the carcinogenic process is likely mediated by its m6A demethylase function, which can affect fundamental signaling pathways such as the MAPK and PI3K/AKT/mTOR pathways [106-107].

FTO can promote neuronal ferroptosis by reducing the expression of SLC7A11 or exacerbate neuronal apoptosis by increasing the expression of ATM, thereby accelerating the progression of PD. In a hepatic ischemia/reperfusion injury (HIRI) disease model, FTO inhibits the stability of mRNAs encoding acyl-CoA synthetase long chain family 4 (ACSL4) and transferrin receptor protein 1 (TFRC) in an m6A-dependent manner to inhibit ferroptosis and improve HIRI [108]. In the 3xTg-AD mouse model, overexpression of FTO downregulates Tsc1 at the mRNA level and alleviates the inhibition of the mTOR pathway, leading to excessive Tau phosphorylation and promoting the progression of AD. Interestingly, upregulation of FTO can enhance the expression of hsa_circ_0112136, activating the PI3K/AKT/mTOR pathway, and promoting the progression of gastric cancer (GC) [109]. Conversely, in prostate cancer (PC), FTO enhances the expression of miR-139-5p in an m6A-dependent manner, resulting in the inactivation of the PI3K/Akt/mTOR signaling pathway and inhibiting the progression of PC [110].

FTO inhibits PGC-1α, thereby limiting mitochondrial biogenesis and activating the NOX4/NADPH oxidase complex, which exacerbates vascular inflammation and plaque formation, thereby accelerating the progression of atherosclerosis. Interestingly, studies have shown that in a mouse atherosclerosis model, FTO inhibits VSMC aging, and subsequently, by stabilizing the MIS12 protein, it slows the progression of atherosclerotic plaques [70]. One report also suggests that FTO inhibits clear cell renal cell carcinoma (ccRCC) through PGC-1α. In ccRCC, FTO functions as a key tumor suppressor. Specifically, FTO expression is downregulated in ccRCC tissues, and low FTO levels are associated with worse tumor progression and patient survival. Crucially, restoring FTO in Von Hippel-Lindau-deficient cells can rescue mitochondrial function, trigger oxidative stress and ROS generation, and suppress tumor growth. This protective effect occurs through an FTO-mediated reduction in m6A modifications of the PGC-1α mRNA, resulting in increased PGC-1α expression [111].

In conclusion, this study highlights the multifaceted role of FTO in the pathogenesis of non-cancerous diseases, emphasizing its structural properties and potential as a pharmacological target. Although FTO-directed therapies are not yet in routine clinical use, accumulating evidence from molecular docking analyses and translational research supports its status as a candidate druggable protein. The therapeutic agents involved in the docking analyses conducted herein are classic drugs primarily used for the treatment of non-cancerous diseases. FTO serves as a key regulatory molecule in the context of these diseases and exhibits strong binding affinity when interacting with these conventional therapeutic agents. These drugs may exert their therapeutic effects at least in part through FTO, via other molecular targets, or even through a broader molecular network involving FTO and its interacting partners. However, attention should also be paid to the ethical implications of FTO-targeted therapy. In animal experiments, all analyses must be carried out in accordance with relevant guidelines and ethical oversight, particularly when evaluating gene editing or CNS delivery systems, thereby helping to ensure accuracy and reliability.

## Data Availability

All data are included in the manuscript.
